# A Recombinant Porcine Epidemic Diarrhea Virus with Multiple S2 Subunit Mutations from China: Isolation, Genetic Characterization, and Pathogenicity Analysis

**DOI:** 10.3390/microorganisms14010242

**Published:** 2026-01-21

**Authors:** Nana Yan, Jingru Xu, Yuqi Li, Sisi Fan, Shuqi Qiu, Linjie Huang, Xiaoziyi Xiao, Yuting Liao, Weiye Lin, Bo Dong, Ailing Dai, Kewei Fan

**Affiliations:** 1 Fujian Provincial Key Laboratory for Prevention and Control of Livestock Infectious Diseases and Biotechnology, College of Life Sciences, Longyan University, Longyan 364012, China; yan@lyun.edu.cn (N.Y.); xvrry07@163.com (J.X.); liyuqi0524@126.com (Y.L.); 18750308680@163.com (S.F.); 15159013956@163.com (S.Q.); hlinjie1112@163.com (L.H.); xiaoziyi20040423@163.com (X.X.); lyt15878345978@163.com (Y.L.); 18738765066@163.com (W.L.); bodong1983@lyun.edu.cn (B.D.); 2 College of Animal Science, Fujian Agriculture and Forestry University, Fuzhou 350002, China

**Keywords:** PEDV, isolation, mutations, S2, pathogenicity

## Abstract

Porcine epidemic diarrhea virus (PEDV) is a major cause of fatal diarrhea in piglets. The continuous emergence of new variants, driven by recombination and mutation, poses a persistent global threat to the swine industry, resulting in significant economic losses. Therefore, ongoing surveillance of PEDV evolution is critical. In this study, we isolated a novel PEDV strain, designated PEDV/FJLY202201, from experimental intestinal samples collected from a diarrheal piglet in Fujian, China, and sequenced its complete genome. Complete genome analysis, phylogenetic analysis, and recombination analysis were conducted. Results showed that PEDV/FJLY202201 was a recombinant strain derived from two recombination events between G2a and G2b strains, with three breakpoints located in the ORF1b, Domain 0 (D0) and S2 subunit, respectively. Notably, multiple mutations were identified in the S2 subunit, a finding that has been rarely reported before. Furthermore, following challenge with the PEDV/FJLY202201 strain, 3-day-old piglets exhibited severe diarrhea, sustained a 30.35% weight loss, and reached 100% mortality, collectively demonstrating its high virulence. These data reveal the complex evolution of PEDV/FJLY202201 and provide a foundation for a better understanding of the genetic evolution and molecular pathogenesis of PEDV.

## 1. Introduction

Porcine epidemic diarrhea (PED) is a highly infectious disease caused by porcine epidemic diarrhea virus (PEDV) [1]. Its clinical signs mainly include diarrhea, vomiting, dehydration, and anorexia, leading to high morbidity and mortality rates in piglets. The first clinical description of PEDV was reported in the UK in 1971 and subsequently spread rapidly throughout Europe before reaching Asian countries [2]. In 2010, a large-scale PEDV outbreak occurred in southern China, even among herds vaccinated with the attenuated CV777 strain [3]. This event marked the start of the global spread of new PEDV variants, leading to extensive and recurrent outbreaks [4,5]. These PEDV variants primarily cause acute diarrhea, vomiting, and dehydration in pigs, with severity and mortality being highest in newborn piglets, often reaching 100% [6]. The virus infects intestinal enterocytes, leading to severe villous atrophy, functional impairment of digestive enzymes, and disruption of tight junctions, which results in malabsorption and dehydration [1]. This process is exacerbated by vomiting and anorexia, which further compromise fluid and electrolyte balance [1]. Today, PEDV is the primary cause of piglet diarrhea on farms, inflicting severe economic losses on the Chinese swine industry [1].

Based on genetic differences, PEDV is classified into two major genotypes: classical (G1) and variant (G2) strains, with the latter further subdivided into G2a, G2b, and G2c [7]. Some classification systems also recognize S-INDEL strains as a distinct category [6]. Currently, G2 strains, which generally exhibit higher pathogenicity than G1 strains, are the predominant strains circulating globally, causing substantial economic losses to the pig farming industry Country [6,8,9].

PEDV is an enveloped, single-stranded RNA virus belonging to the family *Coronaviridae* [10]. Its genome is approximately 28 kb in length and contains at least seven open reading frames (ORFs) that encode the following proteins in the order from the 5′ to 3′ end: ORF1a, ORF1b, spike (S), ORF3, envelope (E), membrane (M), and nucleocapsid (N) [11]. Similarly to other coronaviruses, the PEDV S protein is crucial to viral entry, cell tropism, immunogenicity, and pathogenicity, and it serves as the primary target for neutralizing antibodies [10]. As one of the most variable regions within the PEDV genome, the S gene is a key genetic marker for assessing viral genetic diversity [12,13]. Numerous PEDV strains have been documented to harbor amino acid mutations in their S proteins, which can significantly alter the virus’s pathogenicity, cell tropism, and antigenicity [7,14,15,16]. The S protein consists of two subunits, the N-terminal S1, which is responsible for receptor binding, and the C-terminal S2, which contains fusion peptides that mediate membrane fusion during viral entry [10].

Recombination is also a crucial driver for PEDV evolution, which increases the complexity of the genetic variants that can alter viral pathogenicity and transmissibility [7]. These changes pose significant challenges to the prevention and control of PED. Recombination was principally observed in several major regions of PEDV, including the S1 subunit and the N gene [17]. Research revealed that China hosts the most diverse PEDV lineage, with frequent introduction of PEDV from countries such as South Korea, Japan, and the United States [10,18]. There is evidence indicating that the initial introduction of PEDV into China may have occurred through South Korea, and it may be associated with a recombination event [18].

In this study, we isolated a novel, highly virulent PEDV strain, designated PEDV/FJLY202201, from a diarrheal piglet in Fujian, China. Genomic analysis identified it as a recombinant derived from G2a and G2b strains, with breakpoints in the ORF1b, D0, and S2 subunit, respectively, and multiple previously unreported mutations were identified in the S2 subunit. Challenge experiments in 3-day-old piglets resulted in severe diarrhea and 100% mortality, confirming its high virulence and highlighting the ongoing complex evolution of PEDV.

## 2. Materials and Methods

### 2.1. Ethics Statement

The clinical samples of piglets used in this study were all collected in strict accordance with the Animal Ethics Procedures and Guidelines of the People’s Republic of China. All animal handling and experimental procedures were approved by the Animal Ethics Committee of Longyan University (permit no. LY2024015L).

### 2.2. Virus Isolation and Identification

Intestinal tissues were collected from piglets with severe diarrhea from a pig farm in Fujian, China, in 2022. These samples were diluted with an equal amount of phosphate-buffered saline (PBS), subjected to three freeze–thaw cycles, homogenization, and then centrifuged at 8000 rpm for 10 min at 4 °C. The supernatant was filtered through a 0.22 mm pore size filter. Vero cells (China Center for Type Culture Collection, Wuhan, China) were cultured in Dulbecco’s Modified Eagle Medium (DMEM; Gibco, Grand Island, NY, USA) with 10% fetal bovine serum (FBS, Gibco, USA) and antibiotics (100 U/mL penicillin, 100 μg/mL streptomycin, Gibco, USA) at 37 °C in a humidified incubator with 5% CO_2_. Upon infection, 100 μL of the supernatant was mixed with trypsin (10 μg/mL) and inoculated onto monolayers of Vero cells (China Center for Type Culture Collection, Wuhan, China) grown in a 60 mm cell culture dish (EasyDish, Thermo Scientific, Shanghai, China) to isolate PEDV. The cells were observed daily and were normally maintained for up to 3 days. When approximately 80% of cells showed cytopathic effect (CPE), the cell cultures were collected. After one freeze–thaw cycle, the harvested supernatant was inoculated onto newly prepared Vero cells for subculture and propagated continuously. Otherwise, cell cultures were collected, and blind passages were performed until a typical CPE appeared (CPEs were observed from the fifth generation of blind passage). For plaque purification, monolayers of Vero cells were inoculated with this strain. After incubation for 2 h, the cells were overlaid with 1.5% agarose. Plaques were stained with neutral red dye at 48 h post-inoculation (hpi). For electron microscopy, Vero cell monolayers inoculated with the purified virus were fixed with 2.5% glutaraldehyde at 12 hpi, after which the medium was discarded and the cells were gently scraped off. The cells were then observed using a transmission electron microscope (FEI, Brno, Czech Republic). All steps were performed at room temperature (RT). Virus titer was measured using the Reed-Muench method [19].

### 2.3. Reverse Transcription PCR (RT-PCR) Analysis and Complete Genome Sequencing

Viral genomic RNA was extracted from cell culture supernatant of PEDV-infected Vero cells using a viral nucleic extraction kit (Tiangen Biotech Co., Ltd., Beijing, China) according to the manufacturer’s instructions. The extracted RNA was then reverse-transcribed into cDNA using HiScript Reverse transcriptase (Vazyme, Nanjing, China). Amplification of the PEDV-M fragment was conducted with a specific primer pair targeting the M gene (forward, 5′-TGAAACAGACGCGCTTCTCA-3′; reverse, 5′-TGAGTAGTCGCCGTGTTTGG-3′). The amplification products were analyzed by electrophoresis on a 2% agarose gel. The extracted PEDV/FJLY202201 RNA samples were sent to Guangdong Meige Gene Technology company for complete genome sequencing. The complete genome sequence of the PEDV/FJLY202201 strain has been deposited in GenBank under accession number PQ288283.1.

### 2.4. Immunofluorescence Assay (IFA)

Vero cells grown in a 6-well plate were infected or mock-infected with PEDV/FJLY202201 for 24 h. Subsequently, the cells were fixed with 4% paraformaldehyde for 30 min and permeabilized with 0.2% Triton X-100 for 10 min at room temperature (RT), followed by three washes with PBS. After blocking with 5% bovine serum albumin (BSA) for 2 h at RT, the cells were washed three times with PBS. The cells were first incubated with a mouse anti-PEDV N protein monoclonal antibody (Keepseeking Biotechnology, Guangzhou, China) for 1.5 h at 37 °C, and then with FITC-conjugated goat anti-mouse secondary antibody (Boster Biological Technology, Wuhan, China) for 30 min at 37 °C. Finally, cell nuclei were stained with DAPI (Boster, Wuhan, China) at RT, followed by three washes with PBS. Fluorescence images were acquired using an inverted fluorescence microscope (IX51, Olympus, Tokyo, Japan). The assay was independently repeated three times with similar results.

### 2.5. Phylogenetic and Recombination Analysis

The genome sequences of 27 PEDV references strains deposited in the GenBank database were used for comparative sequence analysis (Table 1). The phylogenetic analysis was conducted using the neighbor-joining method in MEGA 12 software, and the reliability of the tree was evaluated by bootstrapping through 1000 replicates [20].

The putative breakpoints and recombination events in PEDV/FJLY202201 were analyzed using the recombination detection program (RDP 4.10), and seven methods (RDP, BootScan, GENECONV, Chimaera, MaxChi, SiScan, and 3Seq) were employed. A recombination event was identified when at least three of the seven methods reported recombination signals in RDP 4.10, with the highest acceptable *p* value being 0.01 [21]. Additionally, potential recombination events were further analyzed using the SimPlot 3.5.1 software [22].

### 2.6. Animal Experiment

A total of eight 3-day-old piglets were purchased from a commercial pig farm. Prior to the experiment, piglets were tested and confirmed to be negative for PEDV, transmissible gastroenteritis virus (TGEV), porcine deltacoronaviruses (PDCoV), and porcine enteric alphacoronavirus (PEAV). Piglets were randomly allocated to either a challenge group or a control group (*n* = 4 per group) and housed in separate, individually ventilated rooms. They were fed with the Milk Replacer for Piglets (Fujian Province Putian Xinxingda Feedstuff Corporation, Fuzhou, China) every 4 h. Piglets in the challenge group were orally inoculated with 3 mL of PEDV/FJLY202201 (10^5.23^ TCID_50_/mL), and piglets in the control group were inoculated with 3 mL of DMEM in the same route. Daily observations were made to monitor and record clinical signs including diarrhea, vomiting, anorexia, body weight, body temperature, and mortality.

### 2.7. Histopathology and Immunohistochemistry (IHC)

Piglets that died during the study were necropsied immediately. All the surviving piglets in both the challenge and control groups were euthanized by anesthesia at 7 dpi for post-mortem examination. Tissues of duodenum, jejunum and ileum collected from the challenge and control groups were collected, fixed in 10% formalin for at least 24 h at RT, and embedded in paraffin. Fixed tissues were cut into a thickness of 5 µm slices using a microtome (Leica, Shanghai, China). After deparaffinized with xylene and ethanol, slices were then stained with hematoxylin and eosin (H&E; Beyotime, Shanghai, China). For IHC staining, slices were treated with 3% H_2_O_2_, followed by blocking with 0.5% PBS. Subsequently, the slices were incubated with mouse anti-PEDV N protein monoclonal antibody (Keepseeking Biotechnology, Guangzhou, China) overnight at 4 °C and then treated with horseradish-peroxidase-conjugated anti-mouse immunoglobulin G (IgG) antibody (Vector Laboratories, Newark, CA, USA) at room temperature for 1 h.

### 2.8. Biosafety and Biocontainment Procedures

All experiments involving the live PEDV FJLY202201 strain were conducted under Biosafety Level 2 (BSL-2) containment conditions, in accordance with the institutional biosafety guidelines. A comprehensive activity-specific risk assessment was performed prior to the study. All procedures capable of generating aerosols or droplets (e.g., virus handling, sample processing) were performed within a Class II biological safety cabinet (ThermoFisher Scientific, Shanghai, China). Personnel wore appropriate personal protective equipment, including lab coats, gloves, eye protection, and masks. All surfaces and equipment were decontaminated with disinfectants effective against coronaviruses (e.g., sodium hypochlorite solutions).

### 2.9. Statistical Analysis

Statistical analysis was performed with the unpaired *t*-test or the Logrank test. Data were reported as Mean ± Standard Error of the Mean (SEM). *p* values of less than 0.05 were considered statistically significant.

## 3. Results

### 3.1. Isolation and Identification of the PEDV/FJLY202201 Strain

**Figure 1:**
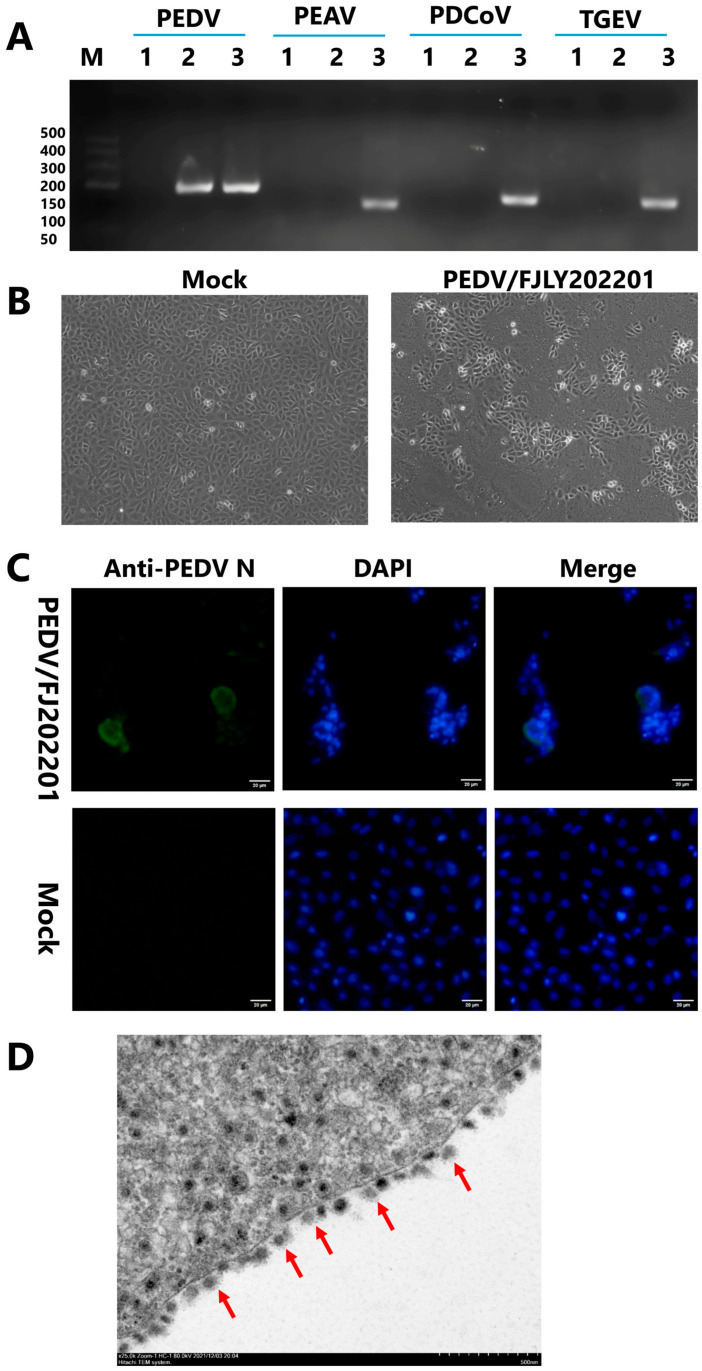
Identification and isolation of the PEDV/FJLY202201 strain. (**A**) Detection of PEDV and confirmation of the absence of PEAV, PDCoV, TGEV in small intestinal tissue samples. Viral genomic RNA was extracted using a viral nucleic extraction kit (Tiangen Biotech Co., Ltd., Beijing, China). The presence of PEDV, PEAV, TGEV, and PDCoV were assessed by PCR using specific primers (Table 2). Lane 1: negative control; lane 2: small intestine tissue sample from diarrheic piglets; lane 3: positive control for PEDV, PEAV, PDCoV, or TGEV, respectively. (**B**) Processed PEDV/FJLY202201-infected intestinal contents were inoculated into Vero cells, and CPEs were observed from the fifth generation of blind passage using light microscopy (100×). (**C**) IFA of Vero cells infected with the PEDV/FJLY202201 strain. Infected cells showed specific reactivity to a PEDV N protein antibody, as indicated by green fluorescence. Scale bar, 20 µm. The assay was independently repeated three times with similar results. (**D**) Transmission electron microscopy image of PEDV/FJLY202201-infected Vero cells. Viral particles exhibited a spherical morphology with surface projections characteristic of coronaviruses Viral particles appeared spherical with a coronavirus-like surface structure (indicated by red arrows). Scale bar, 500 nm.

To determine the causative agent of the diarrhea outbreaks, pigs with severe diarrhea were euthanized, and intestinal contents were collected. Diarrhea-related viruses including PEDV, PEAV, TGEV, and PDCoV were tested by PCR, and only PEDV was detected (Figure 1A), indicating that severe diarrhea was likely caused by PEDV infection. To isolate this PEDV strain, the processed intestinal contents were inoculated into Vero cells, and CPEs were observed from the fifth generation of blind passage. Compared with the mock-infected cells, infected Vero cells showed CPEs characterized by syncytium formation, cell rounding, enlargement, and detachment (Figure 1B). Vero cells were further examined by IFA, and results showed that this strain specifically reacted to the PEDV N protein antibody (Figure 1C). Under transmission electron microscopy, the viral particles appeared spherical with a coronavirus-like surface structure and a diameter of approximately 100 nm (Figure 1D). These results confirmed that the isolated strain was PEDV, and it was designated as PEDV/FJLY202201. The virus titer was determined to be 10^5.23^ TCID_50_/mL on Vero cells.

### 3.2. Complete Genome Sequence Analysis and Phylogenetic Characterization of PEDV/FJLY202201

The complete genome sequence of the PEDV/FJLY202201 strain was identified to be 28,035 nucleotides (GenBank accession number: PQ288283.1). The genomic organization of this strain was consistent with other PEDV strains, encoding four structural proteins (S, E, M, and N proteins) along with non-structural proteins. The complete genome sequence from 5′ to 3′ is organized as: 5′ untranslated region (5′UTR, nucleotide 1–292); ORF1a (nucleotide 293–12,646); ORF1b (nucleotide 12,811–20,637); S gene (nucleotide 20,634–24,791); ORF3 (nucleotide 24,791–25,465); E gene (nucleotide 25,446–25,676); M gene (nucleotide 25,684–26,364); N gene (nucleotide 26,376–27,701); and 3′untranslated region (3′UTR, nucleotide 27,702–28,035). Comparative sequence analysis showed that the nucleotide sequence homology between PEDV/FJLY202201 and 27 PEDV reference strains ranged from 96.2 to 99.0%. Specifically, the identity ranges were 96.2–97.3%, 96.6–96.8%, 98.6–99.0%, 98.0–98.7%, 98.0–98.2% when compared with G1a (e.g., CV777 and virulent-DR13), G1b (e.g., SD-M and JS2008), G2a (e.g., CH-SX-2016 and GD-B), G2b (e.g., AJ1102), G2c (e.g., OH851) reference strains, respectively. Among them, PEDV/FJLY202201 shared the highest similarity with the G2a strain PEDV-CH-SX-2016, which is a virulent G2 strain found in Shanxi, China [7]. The homology of the S gene was also analyzed, and the nucleotide identities between PEDV/FJLY202201 and the 27 strains was 96.6–96.8%, 96.2–97.3%, 98.6–98.7%, 98.0–99.0%, 98.0–98.2% when compared with G1a, G1b, G2a, G2b and G2c reference strains, respectively.

**Figure 2:**
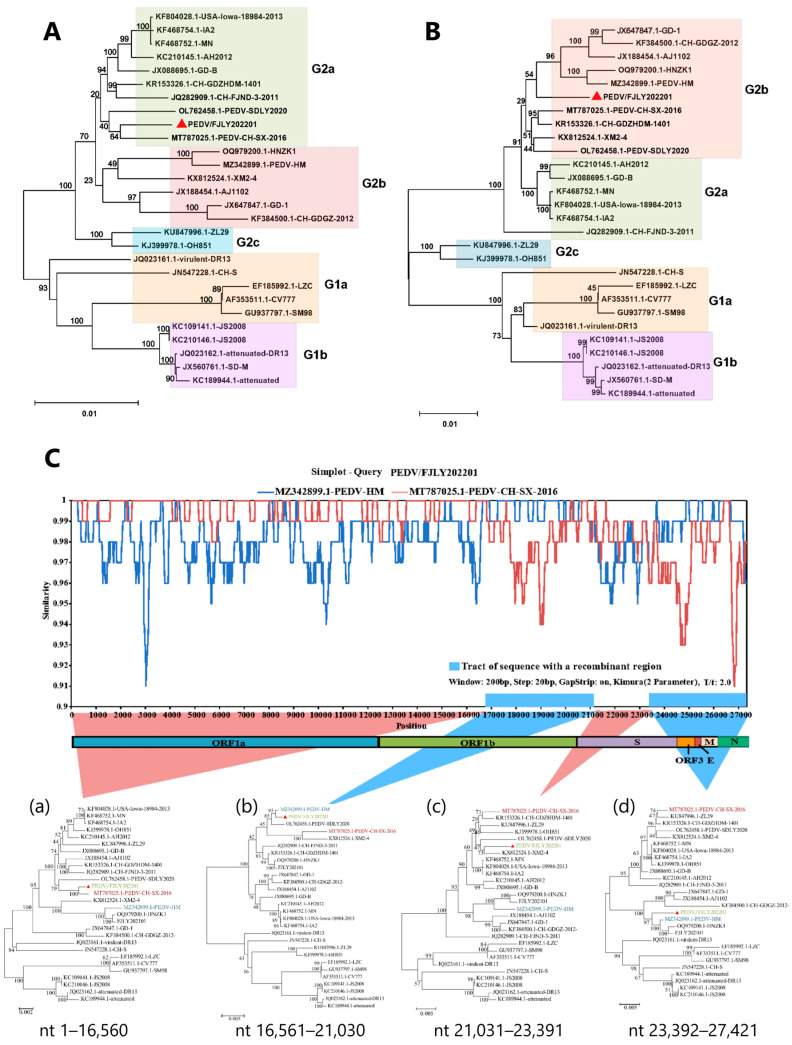
Phylogenetic and recombination analysis of PEDV/FJLY202201. (**A**,**B**): Phylogenetic analysis based on the complete genome (**A**) and S gene (**B**) of PEDV/FJLY202201 and 27 reference strains using the neighbor-joining method in MEGA 12 software. The PEDV/FJLY202201 strain is indicated by a red triangle. (**C**) SimPlot analysis (upper) and statistically incongruent phylogenetic analysis (lower) of the complete genome of PEDV/FJLY202201. In the analysis, PEDV/FJLY202201 was used as the query strain, with PEDV-HM (blue) and PEDV-CH-SX-2016 (red) as the reference strains. The *Y*-axis shows the nucleotide similarity (%) between the query strain and reference strains, and the *X*-axis shows the genome position (nucleotide). (**a**–**d**) phylogenetic trees based on the nucleotide sequences of the regions separated by three breakpoints in PEDV/FJLY202201. The PEDV/FJLY202201 strain is colored in green, the PEDV-CH-SX-2016 strain is colored in red, and the PEDV-HM strain is colored in blue.

To investigate the evolution of PEDV/FJLY202201, phylogenetic trees were constructed based on the whole genome and the S gene sequences, respectively, using the 27 representative strains as reference. The phylogenetic tree based on the complete genomes revealed that PEDV/FJLY202201 belonged to the G2a subgroup, was closely related to the Chinese strains PEDV-CH-SX-2016 and PEDV-SDLY2020, and was distinct from the classical strain CV777 (Figure 2A). Interestingly, phylogenetic analysis of the S gene showed that PEDV/FJLY202201 clustered within the G2b subgroup and formed a new branch (Figure 2B). Based on these findings, we postulated that potential recombination events may have occurred in the PEDV/FJLY202201 strain.

### 3.3. Recombination Analysis

Recombination can lead to significant changes in the genetic diversity of PEDV [7]. To identify possible recombination events, a whole genome recombination analysis between PEDV/FJLY202201 and 27 reference strains was performed using the RDP 4.0 software, which incorporates six detection programs. The analysis revealed three putative recombination breakpoints, one in ORF1b (nt 16,560) and two in the S1 (nt 21,030) and S2 (nt 21,030) subunits of the S protein (Table 3). The putative major parent strain of the recombinant regions was PEDV-HM, belonging to the G2b subgroup, while the putative minor parent was PEDV-CH-SX-2016, belonging to the G2a subgroup. These results were further supported by SimPlot analysis and statistically incongruent phylogenetic trees. As shown in Figure 2C, three breakpoints separated the genome of PEDV/FJLY202201 into four regions: region a (nt 1–16,560), region b (nt 16,561–21,030), region c (nt 21,031–23,391), and region d (nt 23,392–27,421). The crossover regions identified by SimPlot were consistent with the results from the RDP 4.0 analysis, suggesting two putative recombination events. Region a of PEDV/FJLY202201 strain was almost identical to that of CH-SX-2016 strain, and regions b and c were from PEDV-HM. Also, phylogenetic trees based on the nucleotide sequences of region a of PEDV strains showed that PEDV/FJLY202201 clustered closely with strain PEDV-CH-SX-2016, and regions b and d of PEDV/FJLY202201 were close to PEDV-HM strain. Taken together, these findings demonstrate that the evolution of PEDV/FJLY202201 strain resulted from two recombination events between G2a and G2b subgroups.

### 3.4. Multiple Mutations in the S2 Subunit of PEDV/FJLY202201

**Figure 3:**
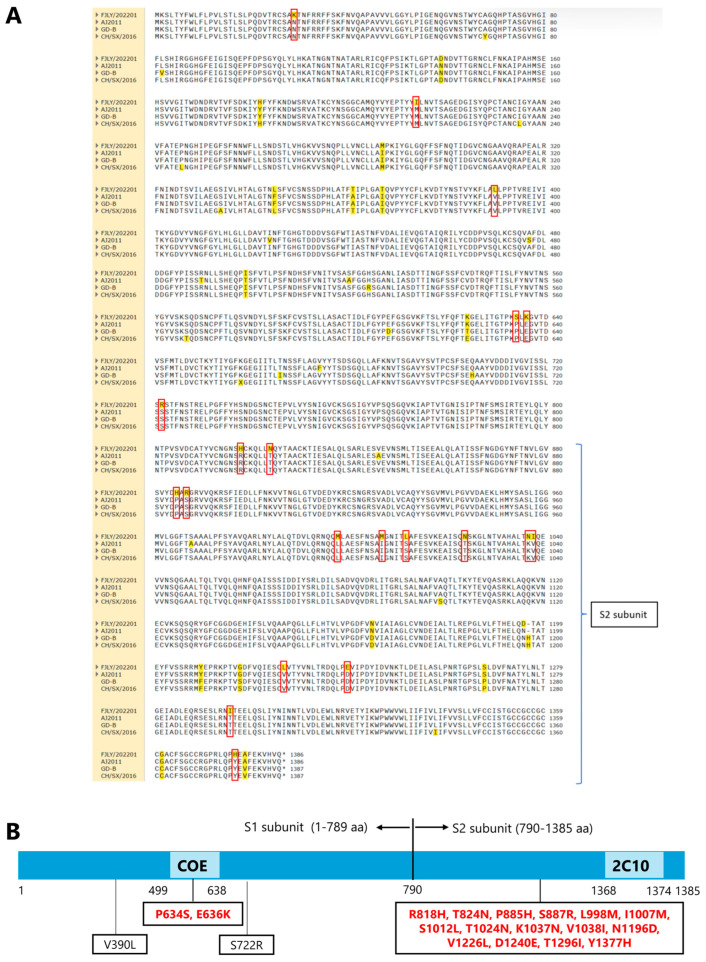
Analysis of amino acid differences in the S protein between PEDV/FJLY202201 and three reference strains (AJ2011, GD-B, and CH/SX/2016). (**A**) Sequence alignment of the S protein from PEDV/FJLY202201 with that of three reference strains, performed using MEGA 12 software. Amino acid substitutions are highlighted in yellow, and unique mutations are framed in red frames. (**B**) Distinct amino acid variations in the S protein of PEDV/FJLY202201 are framed in boxes. Mutations in the COE domain and the S2 subunit are highlighted in red. The numbers at the bottom represent the amino acid positions.

The S gene is a crucial virulence factor of PEDV, and it is often used as a molecular marker for determining the genetic relationships among various strains of PEDV [23]. In this study, the S gene of PEDV/FJLY202201 strain was 4158 bp in length, encoding a 1385-aa protein. To further characterize the genetic variation in the S gene of PEDV/FJLY202201 strain, the amino acid sequence of the S protein was compared with those of vaccine strains AJ1102 and GD-B in the G2b subgroup, and the most similar strain CH-SX-2016. Sequence alignment revealed multiple amino acid substitutions exhibited in PEDV/FJLY202201 strain. Compared to AJ1102, GD-B, and CH-SX-2016 strains, there were 35, 39, and 36 amino acid substitutions in the S protein of PEDV/FJLY202201, respectively (Figure 3A). Previous studies have demonstrated four major neutralizing epitopes that present on the surface of the S protein: CO-26K Equivalent (COE, 499–638 aa), Specific Site 2 (SS2, 748–755 aa), Specific Site 6 (SS6, 764–771 aa) and 2C10 (1368–1374 aa) [16]. A total of 19 unique amino acid substitutions were found in the S gene of PEDV/FJLY202201. Among these, two amino acid substitutions (*P634S*, *E636K*) were in the COE neutralizing epitope, and 15 amino acid substitutions were in the S2 subunit: *R818H*, *T824N*, *P885H*, *S887R*, *L998M*, *I1007M*, *S1012L*, *T1024N*, *K1037N*, *V1038I*, *N1196D*, *V1226*L, *D1240E*, *T1296I*, *Y1377H* (Figure 3B). These results showed a concentration of mutations in the S2 subunit.

### 3.5. Pathogenicity of PEDV/FJLY202201 in Piglets

**Figure 4:**
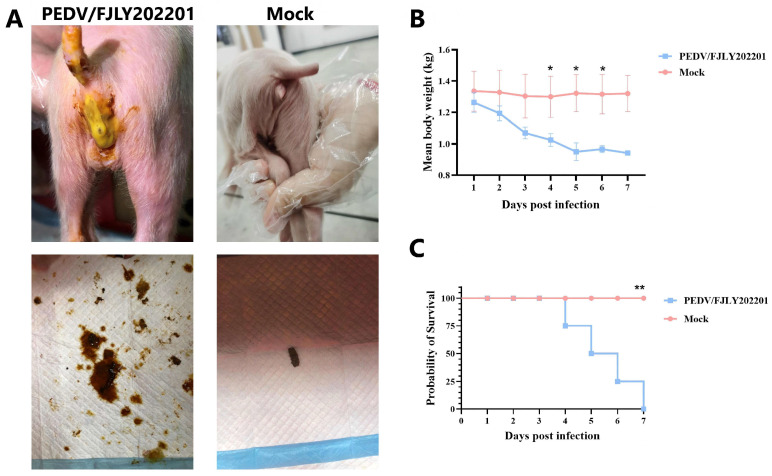
Evaluation of the pathogenicity of PEDV/FJLY202201 in 3-day-old piglets (*n* = 4 for each group). Piglets (*n* = 4 per group) were orally inoculated with PEDV/FJLY202201 or served as mock-infected controls. Piglets were monitored daily for clinical signs, changes in body weight, and mortality. (**A**) Representative clinical images at 2 dpi. Left: PEDV/FJLY202201-infected piglet showing severe diarrhea. Right: mock-infected control piglet. (**B**) Mean body weight changes in piglets following infection. Data are presented as the mean ± SEM. Statistical analysis was performed with unpaired *t*-test in Excel. The statistical significance result at 7 dpi was not presented as there was only one data point in the challenge group. (**C**) Survival rates of piglets in each group. Statistical analysis was performed with Logrank test in Graphpad Prism 10 software. *, *p* < 0.05; **, *p* < 0.01. Figures in (**B**,**C**) were made in the Graphpad Prism 10 software.

To investigate the pathogenicity of PEDV/FJLY202201, 3-day-old piglets were challenged with PEDV/FJLY202201 via oral feeding (*n* = 4), and the piglets inoculated with DMEM were served as the negative control group (*n* = 4). All piglets were monitored daily for clinical signs, including diarrhea, vomiting, and changes in body weight. Gross examination showed that none of the piglets in the control groups developed clinical signs throughout the whole pathogenicity study. In contrast, PEDV/FJLY202201-challenged piglets exhibited PEDV-related clinical signs including diarrhea, vomiting and anorexia at 1 dpi, and experienced severe watery diarrhea thereafter. Notably, challenged piglets developed bloody stools by 2 dpi (Figure 4A). The infected piglets displayed significant weight loss from the 4 dpi, and the mean body weight decreased 30.35% at the end of this experiment (Figure 4B). Furthermore, the infected group exhibited mortality at 4 dpi, reaching 100% mortality by 7 dpi (Figure 4C). These results indicate that PEDV/FJLY202201 is highly pathogenic to newborn piglets.

**Figure 5:**
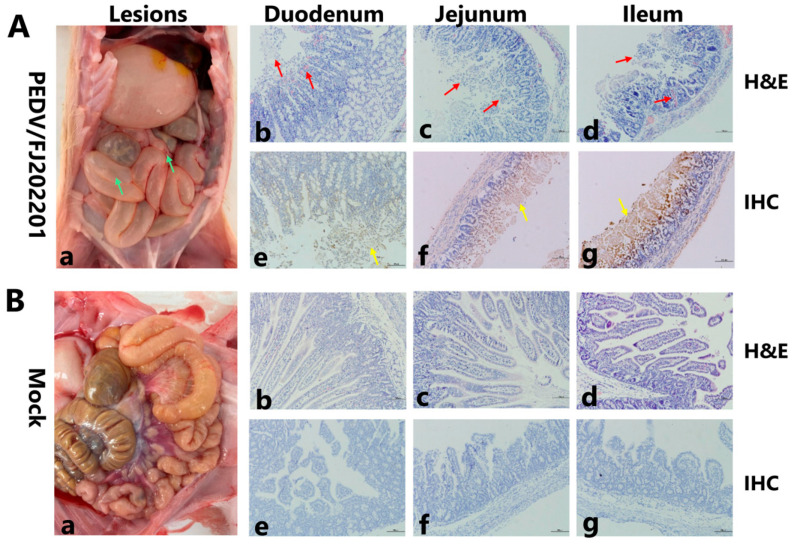
Gross and histopathological examination of small intestinal tissues from piglets inoculated with PEDV/FJLY202201 or DMEM (mock) at 4 dpi. (**A**) PEDV/FJLY202201-challenged group. (**B**) Mock-infected group. (**a**) Gross lesions in the small intestine (indicated by green arrows); (**b**–**d**) H&E staining of duodenum, jejunum, and ileum sections, respectively. Villus atrophy, shortening, and inflammatory cell infiltration are marked by red arrows; (**e**–**g**) IHC staining of duodenum, jejunum, and ileum sections, respectively. PEDV antigens (brown signals) are indicated by yellow arrows. Scale bar, 100 µm.

Necropsy examinations were conducted immediately after the death of infected piglets, and euthanasia and necropsy examinations were conducted on piglets in the control group. The intestinal walls of the challenged piglets were thin and translucent, with gas distension in the intestinal lumen, intestinal hemorrhage, and congestion and hemorrhage of the mesenteric lymph nodes (Figure 5A(a)). In contrast, no significant gross lesions were observed in the intestinal organs of the control piglets (Figure 5B(b)).

Histopathological analysis of the duodenum, jejunum and ileum in the challenge group revealed atrophy and shortening of intestinal villus, along with infiltration of inflammatory cells (Figure 5A(b–d)). In comparison, the control group showed no obvious lesions and revealed a well-preserved tissue structure in the sections of small intestines (Figure 5B(b–d)). Subsequently, the distribution of viral antigens was analyzed by IHC staining, which detected brown signals indicating the presence of PEDV antigens. Results showed that PEDV antigens distributed mainly in the cytoplasm of atrophied villous epithelial cells in the challenge group (Figure 5A(e–g)). In contrast, no viral antigens were detected in the control group (Figure 5B(e–g)). These results demonstrate that PEDV/FJLY202201 is highly pathogenic to piglets (Table 4).

## 4. Discussion

Since the emergence of PED in 1971, numerous PEDV strains with diverse genetic and pathological characteristics have been described [4,7,18]. In particular, G2 subtype strains have become highly prevalent in China since 2010 [7]. Owing to the continuous genetic variation among these strains, sustained monitoring and characterization of circulating viruses in China are essential for developing more effective vaccines. In this study, we successfully isolated a novel recombinant PEDV strain, designated PEDV/FJLY202201, from intestine samples from diarrheal piglets in Fujian, China, and the complete genome was sequenced. We performed phylogenetic analysis, sequence alignment, recombination analysis, and pathogenicity experiments to understand its genetic characteristics and virulence. Results indicated that PEDV/FJLY202201 is a recombinant strain derived from G2a and G2b subgroups, characterized by multiple mutations in the S protein, notably within the S2 subunit. It exhibits high virulence that led to acute diarrhea, vomiting, and 100% mortality in challenged piglets, aligning with the clinical signs of reported prevalent G2 strains [1,6].

Coronaviruses S protein plays a crucial role in antigenicity, pathogenesis, cell and tissue tropism, and cross-species transmission [11]. As is known, the S protein is located on the surface of PEDV and contains the major neutralizing epitopes [16]. Indeed, numerous studies have reported that genetic variations in the newly isolated PEDV strains may lead to changes in the antigenicity and pathogenicity of PEDV [7,9,12,17,23]. Most reports on the variations in the S protein highlight the influence of the mutations on the viral antigenicity, as these mutations were in the neutralizing epitopes in the S1 region, which may reduce the effectiveness of traditional vaccines [10,24]. A recent study conducted a systematic analysis of the molecular characteristics of 1109 PEDV strains circulating in China and found that most mutations were in the S1 region [7].

Interestingly, while we isolated a novel PEDV strain with two mutations within the COE neutralizing epitope in the S1 subunit suggesting its potential impact on antigenicity, the most striking genetic feature is the accumulation of 15 amino acid mutations within the S2 subunit. The S2 subunit forms the stalk of the spike protein and includes the fusion peptide, heptad repeat regions, and transmembrane domain, which are responsible for the critical membrane fusion step after receptor binding by S1 [25]. Historically, the S2 region has been considered more stable than the receptor-binding S1 region due to its conserved function in membrane fusion [25]. The high density of the S2 mutations observed in our strain challenges this assumption and suggests a strong evolutionary driving force. Recent years, many amino acid sites in the S2 subunit have been identified to be critical for cell tropism, including *803L*, *891Q* and *976H* [15,25], suggesting the potential role of the S2 subunit on cross-species transmissibility (15). A report has linked the mutations in the S2 domain of coronavirus to changes in cell tropism [17,26]. In this study, the main genomic characteristic of PEDV/FJLY202201 is the concentrated S2 subunit mutations, suggesting that the virus might be evolving to adapt for interspecies transmission, providing a genetic basis for further research into its phenotypic features. Although in vivo pathogenicity experiments showed a 100% mortality to piglets, in vitro cellular adaptation assays are needed to functionally validate the impact of these mutations.

Recombination also accelerates the genetic evolution process of PEDV, enhancing viral genetic diversity and thereby posing a major challenge to vaccine efficacy [10,17]. In this study, we demonstrated that PEDV/FJLY202201 is recombinant strain derived from two recombination events between G2a and G2b strains, with three breakpoints in the ORF1b region, the D0 domain of the S1 subunit, and S2 subunit. Although intra-G2 subgroup recombination is frequently reported, the occurrence of two sequential recombination events is less common and signifies the complex evolutionary history of this strain. This intricate recombination pattern likely arose from successive co-infections in a single host. The three breakpoints further underscore its genomic complexity. The Pp1b protein encoded by ORF1b is an important non-structural protein involved in the proliferation and synthesis process of the virus [10,24,27]. Recombination in ORF1b may thus affect viral viability and transmissibility [10]. More notably, two breakpoints within the S gene are of particular concern. The D0 domain is implicated in receptor binding and sialic acid interactions, which may affect viral infectivity and cell tropism; it has been identified as a recombination hotspot in Chinese PEDV strains [7]. Furthermore, recombination in the S2 subunit, which mediates membrane fusion, may influence viral entry and cell tropism [25]. Previous reports have indicated that recombination is more likely to occur in the S1 subunit, while S2 subunit is generally considered to be more stable [25]. Collectively, these recombination events are likely to provide a selective advantage, potentially altering cell tropism, pathogenicity, or antigenicity, and may thereby facilitate evasion of herd immunity induced by existing vaccines.

## 5. Conclusions

In conclusion, this study isolated a novel, highly virulent PEDV strain, designated PEDV/FJLY202201. Genomic characterization revealed that it is a complex recombinant, originating from two sequential recombination events between G2a and G2b strains, with three identified breakpoints located in the ORF1b, D0, and S2 regions, respectively. A particularly significant and novel characteristic of PEDV/FJLY202201 is the identification of recombination events and predominant mutations identified within the S2 subunit—a genetic event rarely reported in PEDV. As the S2 subunit is directly responsible for mediating membrane fusion, these variations are strongly predicted to affect cell tropism and could potentially alter host range. The emergence of such a doubly recombinant PEDV strain, characterized by concentrated mutations in the S2 subunit, represents an urgent global threat. It demonstrates the virus’s capacity for rapid evolution, which can outpace existing control measures, undermining global biosecurity and threatening the stability of the swine industry. These findings emphasize the critical need for sustained and intensive surveillance to monitor the real-time evolution of PEDV strains, which is essential for developing effective countermeasures against this continuously evolving pathogen.

## Figures and Tables

**Table 1:** Information of the PEDV reference strains from the Genbank database.

Strain	Accession Number	Location	Collection Year	Genotype
CV777	AF353511	Belgium	1978	G1a
LZC	EF185992	China	2006	G1a
SM98	GU937797	South Korea	2010	G1a
CH-S	JN547228	China	1986	G1a
Virulent-DR13	JQ023161	South Korea	2009	G1a
Attenuated-DR13	JQ023162	South Korea	2002	G1b
JS2008	KC109141	China	2008	G1b
JS2008	KC210146	China	2008	G1b
SD-M	JX560761	China	2012	G1b
PEDV	KC189944	China	2012	G1b
OH851	KJ399978	USA	2014	G2c
ZL29	KU847996	China	2015	G2c
USA/Iowa/18984/2013	KF804028	USA	2013	G2a
IA2	KF468754	USA	2013	G2a
MN	KF468752	USA	2013	G2a
AH2012	KC210145	China	2012	G2a
GD-B	JX088695	China	2012	G2a
CH-GDZHDM-1401	KR153326	China	2014	G2b
CH-FJND-3-2011	JQ282909	China	2011	G2a
PEDV-SDLY2020	OL762458	China	2020	G2b
PEDV-CH-SX-2016	MT787025	China	2016	G2b
HNZK1	OQ979200	China	2021	G2b
PEDV-HM	MZ342899	China	2017	G2b
XM2-4	KX812524	China	2016	G2b
AJ1102	JX188454	China	2011	G2b
GD-1	JX647847	China	2011	G2b
CH-GDGZ-2012	KF384500	China	2012	G2b

**Table 2:** Primers for the amplification of PEDV, PEAV, TGEV, and PDCoV.

Virus	Primer Name	Sequence (5′ to 3′)	Product Size (bp)
PEDV	PEDV-JD180-F	CCTGAAACAGACGCGCTTCT	180
	PEDV-JD180-R	CTTGGCGACTGTGACGAAATT	
PEAV	PEAV-JD120-F	CATGCCAGTCCAGGCCTCAA	120
	PEAV-JD120-R	CACGCTTCCATTCAGGTTTGT	
TGEV	TGEV-JD135-F	GGCCAACGTAAAGAGCTTCCT	135
	TGEV-JD135-F	CCAAGCGTGGTTGGTTTGTT	
PDCoV	PDCoV-JD135-F	TGGGTACATGGAGGTGCATTC	143
	PDCoV-JD135-R	CCATATCCTGTGGCGGATTT	

**Table 3:** Analysis of putative recombination events of isolate PEDV/FJLY202201.

Recombination Events		Major Parent	Minor Parent	*p* Value						
Beginning	Ending			RDP	GENECONV	Bootscan	MaxChi	Chimaera	SiScan	3Seq
16,560	21,030	PEDV-HM	PEDV-CH-SX-2016	1.762 × 10^−8^	3.749 × 10^−21^	-	9.337 × 10^−2^	-	-	1.279 × 10^−10^
23,391	27,421	PEDV-HM	PEDV-CH-SX-2016	1.249 × 10^−30^	3.749 × 10^−21^	-	2.357 × 10^−12^	3.844 × 10^−18^	6.306 × 10^−23^	9.264 × 10^−18^

A whole genome recombination analysis between PEDV/FJLY202201 and 27 reference strains was performed using the RDP 4.0 software. Potential recombination events were identified based on strong *p* value (*p* < 10^−10^).

**Table 4:** Summary of clinical signs, gross lesions, and histopathological findings in PEDV/FJLY202201-challenged piglets.

Parameter	PEDV/FJLY202201-Challenged Group	Control Group
Clinical signs	Diarrhea, vomiting, anorexia, bloody stool, and significant weight loss.	No clinical signs observed throughout the study.
Gross lesions	Thin and transparent intestinal walls, gas distension in the intestinal lumen, intestinal bleeding, and congestion/bleeding of mesenteric lymph nodes.	No significant gross lesions were observed
Histopathology	Severe atrophy and shortening of intestinal villi, and inflammatory cell infiltration.	Well-preserved tissue structure with no obvious lesions.
IHC staining	PEDV antigens (brown signals) were detected in the cytoplasm of atrophied villous epithelial cells.	No viral antigens were detected.

## Data Availability

The original contributions presented in this study are included in the article. Further inquiries can be directed to the corresponding authors.

## Abbreviations

The following abbreviations are used in this manuscript:

BSA	Bovine serum albumin
BSL-2	Biosafety level 2
COE	CO-26K equivalent
CPE	Cytopathic effect
D0	Domain 0
H&E	Hematoxylin and eosin
HRP	Horseradish peroxidase
IFA	Immunofluorescence assay
IgG	Immunoglobulin G
IHC	Immunohistochemistry
M	Membrane
N	Nucleocapsid
ORF	Open reading frame
PBS	Phosphate-buffered saline
PDCoV	Porcine deltacoronaviruses
PEAV	Porcine enteric alphacoronavirus
PED	Porcine epidemic diarrhea
PEDV	Porcine epidemic diarrhea virus
RT	Room temperature
SEM	Standard error of the mean
S	Spike
SS2	Specific site 2
SS6	Specific site 6
TGEV	Transmissible gastroenteritis virus
